# Interleukins 4 and 21 Protect Anti-IgM Induced Cell Death in Ramos B Cells: Implication for Autoimmune Diseases

**DOI:** 10.3389/fimmu.2022.919854

**Published:** 2022-07-13

**Authors:** Chin Wai Hui, Wai Chung Wu, Shui On Leung

**Affiliations:** SinoMab BioScience Limited, Pak Shek Kok, Hong Kong, Hong Kong SAR, China

**Keywords:** interleukin 4 (IL-4), interleukin 21 (IL-21), hyperactivated B cell, B cell tolerance checkpoint, autoimmune disease

## Abstract

Interleukins 4 (IL-4) and 21 (IL-21) belong to the common gamma chain cytokine family which are highly involved in the progression of autoimmune diseases. While IL-4 is well known to be involved in the suppression of apoptosis of autoreactive B cells, the role played by IL-21 remains unclear. In the current study, we activated the human Burkitt’s lymphoma Ramos B cells with anti-IgM to mimic B cell hyperactivation observed in patients of autoimmune diseases. Consistent with other reported findings, anti-IgM led to the downregulation of proteins involved in B cell survival and proliferation, as well as the activation of caspase 3 activity and DNA damage, resulting in apoptotic cell death after 48-hour treatment. Although both IL-4 and IL-21 reversed anti-IgM-induced apoptosis and cell cycle arrest, they did so *via* different mechanisms: while IL-4 could directly suppress anti-IgM-induced caspase 3 activation and marker indicative of DNA damage, IL-21 could induce B cell proliferation in the presence of anti-IgM. Importantly, IL-21 also suppressed activation induced cell death in human primary B cells. Pre-treatment with clinically validated JAK inhibitors completely reversed the effects of IL-4 and IL-21 to rescue anti-IgM induced cell death and DNA damage. The results indicate the underlying mechanisms of how IL-4 and IL-21 differentially promote survival of hyperactivated B cells and provide hints to treat autoimmune diseases.

## Introduction

The presence of B cell hyperactivity is one of the characteristics associated with the progression of autoimmune disease, including systemic lupus erythematosus (SLE), rheumatoid arthritis (RA), type 1 diabetes (T1B), Sjögren’s syndrome (SS) and systemic/multiple sclerosis (SS/MS) (1–6). In healthy individuals, elimination of self-reactive B cells is well controlled by apoptosis through the activation of pro-apoptotic Bcl-2 family and Fas-mediated process in a CD4(+) T cell-dependent manner during negative selection (7–9). However, expansion of autoreactive B cells happens when the B cell tolerance checkpoints are impaired during autoimmune diseases (10–12). The autoreactive B cells potentially trigger the disease progression through secretion of autoantibodies, presentation of autoantigens, secretion of proinflammatory cytokines, modulation of antigen processing/presentation and the formation of ectopic germinal centers (13). Except genetic mutation, cytokine imbalance is another important factor leading to increased survival and proliferation of autoreactive B cells in these diseases.

Interleukin-4 (IL-4) and interleukin-21 (IL-21) belong to common gamma chain family cytokines which could induce the downstream signaling pathways through dimerization of IL-4/IL-21 receptor and common gamma chain receptor. Both cytokines are secreted by T follicular helper (T_FH_) cells (14, 15) to promote pleiotropic activity in B cells, including proliferation and activation of naive B cells, differentiation into mature and plasma cells, class switch recombination, and formation of germinal centers and memory B cells in human and mice (16–21). Despite the normal functions, IL-4 and IL-21 are highly involved in the progression of autoimmune diseases. The genetic associations between IL-4/IL-21 and autoimmune diseases had been well documented (22–33). Furthermore, elevated IL-4/IL-21 serum levels and increased numbers of IL-4/IL-21 producing T cells were observed in several human autoimmune conditions (34–42). Although *in-vitro* and animal studies have provided clues to explain how IL-4 promotes abnormal active B cell function and survival during disease conditions (43–48), the effect of IL-21 in B cell pathology remains unclear.

In the current study, we demonstrated how IL-4 and IL-21 differentially disrupted the B cell tolerance checkpoint upon anti-IgM activation in Ramos B cells. Ramos is a human Burkitts Lymphoma cell line and could be used as a model for B-cell antigen receptor (BCR) activation by cross-linking the BCR with anti-IgM antibody, which would lead to the induction of cell cycle arrest and apoptosis. Ramos was chosen in the current study because of the following reasons. First, Ramos cells are negative for EBV infection which better mimics the normal primary B cells, given that EBV infection potentially modulates the B cell phenotype (49). Second, more detailed studies of anti-IgM effects on Ramos cells were performed when compared to other B cell lines (50). Third, anti-IgM treatment led to more obvious induction of apoptosis and growth arrest in Ramos cells when compared to other B cell lines (51), thus greatly mimicking the tolerance checkpoint induced apoptosis in hyperactivated primary B cells. We first translated the studies performed in murine B-cell lymphoma cell line CH31 (45) into human Ramos cell line, confirming that IL-4 could similarly reverse the anti-IgM induced apoptosis through cell maintenance in G1 phase, suppression of DNA damage and attenuation of cleaved caspase 3 activity in human system. Interestingly, IL-21 suppressed anti-IgM induced apoptosis *via* a different route, leading to the induction of proliferation and accumulation of intermediate caspase-3 p19 isoform in the Ramos model. Treatment with clinically validated JAK inhibitors blocked STAT6 and STAT3 activation in IL-4 and IL-21 treated Ramos cells respectively and completely abolished rescues of anti-IgM induced cell death. Preliminary results also demonstrated that IL-21 was able to suppress CD40 ligand (CD40L) and anti-IgM induced cell death in human primary B cells during activation. These results suggest that IL-4 and IL-21 could disrupt the B cell tolerance checkpoint and promote the survival of hyperreactive B cells through different mechanisms. The blockade of JAK/STAT pathway might serve as the strategy to treat autoimmune diseases associated with IL-4 and IL-21 upregulation.

## Materials and Methods

### Cells and Reagents

The human Burkitts Lymphoma cell line Ramos (CRL-1596™) and human T lymphoblast cell line Jurkat (Clone E6-1, TIB-152™) were purchased from ATCC. Low passage (20<) Ramos cells was cultured in complete RPMI 1640 Medium (#A1049101, ATCC modification, ThermoFisher Scientific, Waltham, MA, USA), supplemented with 100 U/ml penicillin, 100 μg/ml streptomycin (15140122, ThermoFisher Scientific), and 10% fetal bovine serum (FBS, A3160801, ThermoFisher Scientific. Low passage of Jurkat cells (20<) was cultured in the same complete medium but without antibiotics. Cells were sub-cultured at 5x10^5^ cells/ml and maintained at 37°C in a humidified 5% CO_2_ atmosphere. Recombinant human IL-2 (#11848-HNAH1-E), IL-4 (#11846-HNAE), IL-7 (#11821-HNAE), IL-9 (#11844-H08B), IL-15 (#10360-HNCE) and IL-21 (#10584-HNAE) were obtained from Sino Biological (Beijing, China). JAK inhibitors Tofacitinib (Tof, #4556), Ruxolitinib (Rux, #7064) and Ritlecitinib (Rit, #6506) were purchased from Tocris (Abingdon, United Kingdom). AffiniPure Goat Anti-Human IgM, Fc5μ fragment specific (#109-006-129) was obtained from Jackson ImmunoResearch (West Grove, USA).

### Quantitative Real-Time PCR

For receptor expression studies, Ramos (10^6^ cells/ml) and Jurkat (10^6^ cells/ml) cells were harvested for RNA extraction. For STAT driven gene expression studies, Ramos cells (10^6^ cells/ml) were starved in serum-free RPMI1640 medium for 24 hours and then treated with 10 or 50 ng/ml IL-4 or IL-21 for 6 and 24 hours. Total RNA was extracted from Ramos and Jurkat cells by RNAzol (R4533, Sigma, St. Louis, Missouri, USA) following manufacturer’s protocol. All RNA showed 260/280 Ratio of 1.8 to 2.0 as measured by NanoDrop™ OneC microvolume UV-Vis spectrophotometer (701-058108, ThermoFisher Scientific). Reverse transcription was performed in 500 ng of total RNA using PrimeScript RT reagent kit (RR037A, Takara, Beijing, China) and the gene expression was analyzed by quantitative real-time PCR using TB Green Premix Ex Taq (RR420W, Takara) in LightCycler^®^ 480 Real-Time PCR System (Roche, Basel, Switzerland). The primers used were summarized in **Table 1**. Housekeeping gene *GAPDH* was used for normalization.

**Table 1:** Primer lists for *homo sapiens*.

	Forward	Reverse
*IL-2Rα*	ATGGATTCATACCTGCTGATGTGG	CAGGACCCAACTCACTTGGTGTAG
*IL-2Rβ*	ATGGCGGCCCCTGCTCTG	CAGGACCCAACTCACTTGGTGTAG
*IL-2Rγ*	TTTCCTGTTTGCATTGGAAGCC	GTTCCCGTGGTATTCAGTAACAA
*IL-4Rα*	CTGCTCATGGATGACGTGGTCA	GGTGTGAACTGTCAGGTTTCCTG
*IL-7Rα*	ATGACAATTCTAGGTACAACTTTTG	CTAGAATGTCCAGGACCGAGCCTT
*IL-9Rα*	ATGGGACTGGGCAGATGCATCTGG	CTAGAATGTCCAGGACCGAGCCTT
*IL-15Rα*	ATGGCCCCGCGGCGGGCG	GAAAACTGCTCTCACCACCTATGA
*IL-21R*	ATGCCGCGTGGCTGGGCC	CCCTGGACCCCAGGCCAGCTAA
*CD23*	GGTATGCCTGTGACGACATGGA	TTCAGGTCCAAGTTCCGAAGGC
*CASP7*	AGTGACAGGTATGGGCGTTC	CGGCATTTGTATGGTCCTCT
*PRDM1*	AACGTGTGGGTACGACCTTG	CCGCATCCTCCATGTCCATT
*IRF4*	CAGCCCAGCAGGTTCACAACTA	CCTGTCACCTGGCAACCATTT
*GADPH*	GTCTCCTCTGACTTCAACAGCG	ACCACCCTGTTGCTGTAGCCAA

### Treatment Paradigm of Ramos Cells

To investigate the effects of cytokines, Ramos cells (10^6^ cells/ml for western blot and 5x10^5^ cells/ml for other experiments) were treated with IL-4 or IL-21 for an hour before challenge with different concentrations of anti-IgM up to 48 hours. To study the blockade of JAT/STAT pathway, cells were pre-treated with different concentrations of Tofacitinib, Ruxolitinib and Ritlecitinib for an additional hour before the cytokine administration. After the indicated timepoints of anti-IgM challenge, cells were harvested for western blot or flow cytometry.

### Western Blot

Antibodies targeting cyclin A (sc-271682), cyclin D (sc-8396), cyclin E (sc-377100), CDK1 (sc-54), CDK2 (sc-6248), CDK4 (sc-23896) and CDK6 (sc-7961) were purchased from Santa Cruz Biotechnology (Dallas, Texas, USA); AKT (#9272), p-AKT (#4060), p65 (#8242), p-p65 (#3033), STAT3 (#9139), p-STAT3 (#9145), STAT5 (#94205), p-STAT5 (#9359), STAT6 (#5397), p-STAT6 (#56554), total caspase 3 (#9662) from Cell Signaling Technology (Danvers, Massachusetts, USA); γ-H2AX (ab26350) from Abcam (Cambridge, UK); and tubulin (100109-MM05T) from Sino Biological. HRP conjugated goat anti-mouse (#7076) and goat anti-rabbit secondary antibodies (#7074) were purchased from Cell Signaling Technology.

Total proteins were extracted from the Ramos cells in RIPA lysis buffer (#20-188, Millipore, Burlington, Massachusetts, USA) supplemented with Halt™ Protease and Phosphatase Inhibitor Cocktail (#78440, ThermoFisher Scientific) after cytokine treatment for 15 mins or anti-IgM treatment up to 48 hours. After measuring the protein concentrations by Pierce™ BCA Protein Assay Kit (#23225, ThermoFisher Scientific), protein lysate was diluted in NuPAGE™ LDS Sample Buffer (#NP0007, ThermoFisher Scientific) supplemented with 5% 2-mercaptoethanol (#1610710, BioRad) and milliQ water to a desired concentration, boiled at 95°C for 10 mins and stored at -80°C until use. Total protein (20 to 40μg/lane) was separated through electrophoresis and blotted on the nitrocellulose membrane (GE10600001, Sigma). Membrane was blocked with 5% nonfat milk (#1706404, BioRad) diluted in TBST, then incubated with primary antibodies diluted in 5% BSA/TBST at 4°C overnight in a rotating wheel. Next day, membrane was washed with PBST and incubated with secondary antibodies (1:2000) diluted in 5% milk/TBST for an hour at RT. Intensities of protein bands were determined using ECL substrate kit (#34580, ThermoFisher Scientific) in the ChemiDoc Imaging System (BioRad).

### Apoptotic Assay

After 48-hour anti-IgM treatment, Ramos cells were washed once in FACS wash buffer (2% FBS in PBS) and then stained with dead cell apoptosis kits with annexin V (AV) for flow cytometry (#V13242, ThermoFisher Scientific) according to the manufacturer’s protocol. Cells were later stained with propidium iodide (PI) provided in the kit, and the percentages of live, apoptotic and necrotic populations were analyzed by BD FACSLyric™ Clinical Cell Analyzer (BD Biosciences, New Jersey, USA). Annexin V and PI single stained cells were used for correcting fluorescence spillover emissions. Unstained control was performed in cells without dye incubation.

### Cell Cycle Analysis

After 48-hour anti-IgM treatment, Ramos cells were washed once in PBS and drop fixed in 95% ethanol on ice for 2 hours. Fixed cells were re-hydrated in FACS wash buffer and stained with 5 μg/ml PI (P1304MP, ThermoFisher Scientific) in the presence of 100 ug/ml RNase A (#19101, Qiagen, Hilden, Germany) at 37°C for 30 mins. Percentages of cells in sub G0/G1, G1, S and G2-M phases were determined by flow cytometry. Unstained control was performed in cells without PI incubation.

### Mitochondrial Membrane Potential (Δψm) Analysis

After 48-hour anti-IgM treatment, Ramos cells were washed once in FACS wash buffer and stained with tetramethylrhodamine (TMRE, #T669, ThermoFisher Scientific) for 15 min at RT. TMRE is a cell-permeant dye that accumulates in active mitochondria with intact membrane potentials. Percentage of cells with intact Δψm was quantified by flow cytometry. Unstained control was performed in cells without dye incubation.

### WST-8 Proliferative Assay

Ramos cells (50000 cells in 100 μl) were seeded into 96-well plate and stabilized at 37°C for an hour. After 48-hour anti-IgM treatment, 10 μl WST-8 reagent (ab228554, Abcam) was added to each well and incubated for 4 to 8 hours. WST-8 could be reduced by cellular dehydrogenases to an orange formazan product, which the amount of formazan produced is directly proportional to the number of living Ramos cells. Optical density (OD) of each well was measured by absorbance at 450 nm using Varioskan LUX Multimode Microplate Reader (ThermoFisher Scientific). Samples were tested in duplicate wells.

### Primary B Cell Isolation and Treatment

Healthy human peripheral blood mononuclear cell (PBMC) was purchased from iXCells Biotechnologies (San Diego, California, USA). PBMC was cultured at the density of 2E6 cells/ml in the complete RPMI1640 medium. After 24 hours, B cells were purified from PBMC culture through negative selection using human B Cell Isolation Kit II (#130-091-151, Miltenyi Biotec, Bergisch Gladbach, North Rhine-Westphalia, Germany). Purified B cells were resuspended in fresh complete RPMI1640 medium and labelled with CFSE Cell Division Tracker Kit (#423801, Biolegend, San Diego, California, USA) following manufacturer’s protocol. Cells were then seeded into 96-well round bottom plate at the density of 2E5 cells/100µl, and then challenged with 50 ng/ml IL-21 and 1 µg/ml CD40L (#6420-CL/CF, R&D Systems, Minneapolis, USA) or 2 µg/ml anti-IgM. Cells were harvested after 3-day incubation.

### Flow Cytometry Analyses of Purified B Cells

Purified B cells were washed once with FACS wash buffer and then stained with PE anti-CD38 (#555460, BD Biosciences). After 30-min incubation at RT, cells were washed once with FACS wash buffer, resuspended in 0.1 µg/ml DAPI (for accessing cell death) and then analyzed by flow cytometry. Unstained control was performed in cells without CSFE and antibody incubation.

### Data Quantification and Statistical Analyses

For western blot, the signal intensities of protein bands were quantified by ImageJ (National Institutes of Health). Data obtained from flow cytometry were analyzed by Flowjo (version 10, BD). Statistical analyses were performed by Prism (version 7, GraphPad). *Student t-test* was used to indicate difference in mean between two groups. One-way ANOVA was used to determine statistical significance of means among 2 or more groups across the time, cytokine concentration or drug concentration. Two-way ANOVA was used to estimate how the mean of a quantitative variable changes significantly according to the levels of two categorical variables (anti-IgM and cytokine concentrations; cytokine and JAK inhibitor concentrations). p < 0.05 is considered statistically significant for all statistical tests used.

## Results

### IL-4 and IL-21 Stimulated JAK/STAT Pathway in Ramos Cells

**Figure 1:**
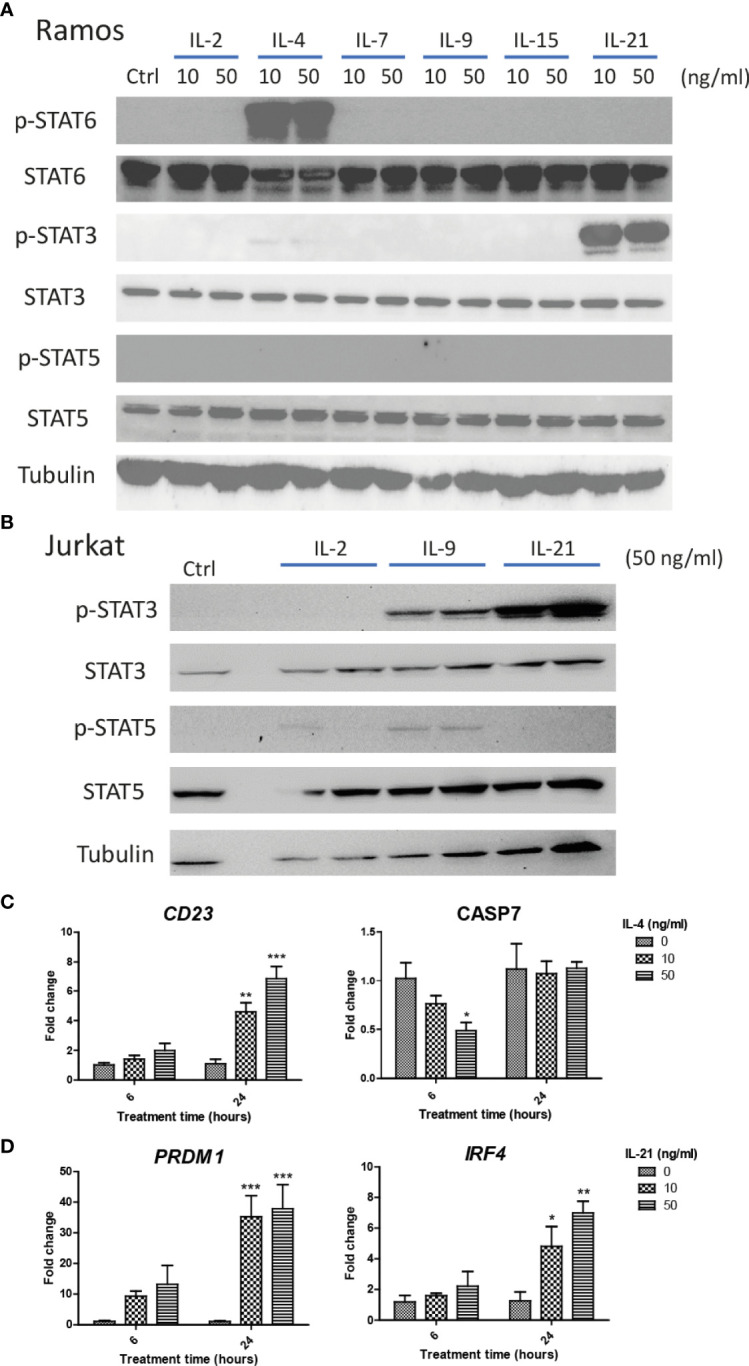
IL-4 and IL-21 activated JAK/STAT pathways and stimulated downstream gene expressions in Ramos B cells. Ramos and Jurkat were treated with γc cytokines for 15 mins and harvested for western blot analyses. **(A)** IL-21 and IL-4 significantly induced STAT3 and STAT6 phosphorylation respectively. No STAT5 phosphorylation was observed in Ramos cells after any cytokine treatments. **(B)** IL-2, IL-9 and IL-21 treatments triggered STAT3 and STAT5 phosphorylation in Jurkat cells. Ramos cells were starved in serum-free RPMI1640 medium for 24 hours and treated with either IL-4 or IL-21 for 6 and 24 hours. **(C)** IL-4 significantly reduced CASP7 and induced CD23 expressions dose dependently at 6 and 24 hours respectively. **(D)** IL-21 significantly induced PRDM1 and IRF4 expressions dose dependently at 24 hours. *p < 0.05, **p < 0.01, ***p < 0.001 as compared to vehicle controls art respective time points by one-way ANOVA, N = 3.

We first investigated if Ramos cells express corresponding receptors to interact with the cytokines used in the current study. Jurkat cells were used as the positive controls as the cells could potentially respond to IL-2, IL-4, IL-9 and IL-21 stimulation (52–55). Comparing the results of qRT-PCR between Jurkat and Ramos cells, Ramos cells showed high expression levels of *IL-2Rα, IL-2Rγ*, *IL-4Ra* and *IL-21R*, low expression levels of *IL-2Rβ* and *IL-7Ra*, and no expressions of other receptor subunits (**Table 2**), suggesting that Ramos cells mainly respond to IL-4 and IL-21 stimulation. Low expression level of *IL-2Rβ* could indicate limited IL-2 induced dimerization of IL-2Rβ and IL-2Rγ, leading to failed stimulation of downstream JAK/STAT pathway. We further confirmed the results through western blot analyses. Phosphorylation levels of two STAT proteins, STAT6 and STAT3 (56, 57), were triggered in Ramos cells after IL-4 and IL-21 treatments respectively (**Figure 1A**). Although STAT3 and STAT5 phosphorylation was observed in Jurkat (**Figure 1B**), no p-STAT5 protein was observed after cytokine treatments in Ramos (**Figure 1A**), indicating that STAT3 and STAT6 are major phosphorylated STAT proteins in Ramos cell line. Biological activities of IL-4 and IL-21 were confirmed by determining the levels of STAT driven gene expressions. Treatment of IL-4 led to increased *CD23* and reduced *CASP7* expressions (**Figure 1C**), while treatment of IL-21 triggered *PRDM1* (*BLIMP-1*) and *IRF4* expressions (**Figure 1D**) in Ramos cells. The data were consistent to other literatures (58–60) and showed that Ramos cells mainly respond to IL-4 and IL-21 treatments within all cytokine groups in the current study.

**Table 2:** CT values for gene expressions of cytokine receptors in Ramos and Jurkat.

	Ramos	Jurkat
*IL-2Rα*	24.49 ± 0.43 **	31.31 ± 1.63
*IL-2Rβ*	32.77 ± 0.19	30.68 ± 0.99
*IL-2Rγ*	23.32 ± 0.37	20.13 ± 1.23 *
*IL-4Rα*	23.85 ± 0.40 **	29.24 ± 1.45
*IL-7Rα*	30.55 ± 0.34	28.15 ± 1.78
*IL-9R*	33.18 ± 0.47	20.13 ± 1.23 ***
*IL-15α*	> 35	> 35
*IL-21R*	24.48 ± 0.53 *	30.25 ± 1.69
*GAPDH*	17.83 ± 0.29	17.82 ± 0.99

Student t-test was used to compare differences between Ramos and Jurkat cells. Same housekeeping GAPDH expression was observed in Ramos and Jurkat groups. ***P<0.001, **p<0.01,*p<0.05, N = 4 for Ramos and N = 3 for Jurkat groups.

### IL-4 and IL-21 Disrupted the B Cell Tolerance Checkpoint and Promoted the Survival of Hyperactivated Ramos Cells

**Figure 2:**
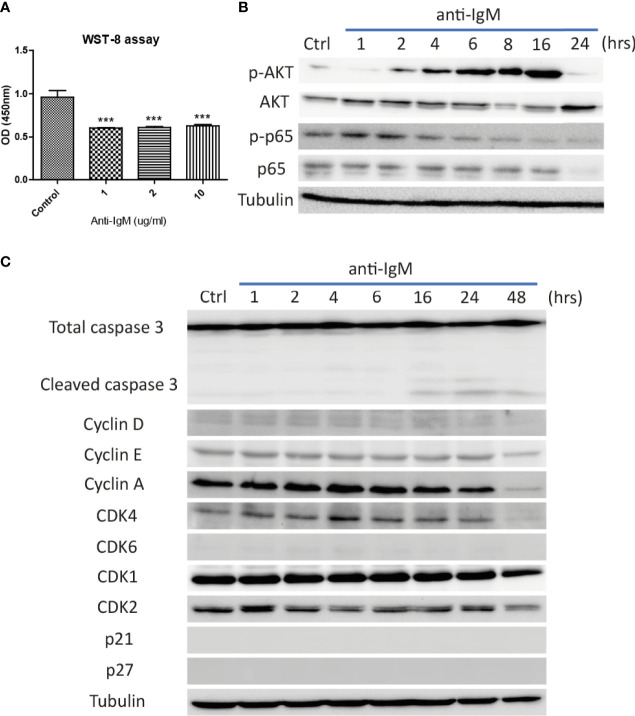
Detrimental effects of anti-IgM on Ramos B cells. **(A)** Anti-IgM treatment significantly reduced proliferation after 48 hours in WST-8 assay. The detrimental effects were driven by **(B)** reduction in p65 signaling and AKT survival proteins, **(C)** activation of caspase 3 activity and downregulation of cell cycle related proteins after incubation of 2 µg/ml anti-IgM. ***p < 0.001 as compared to controls by one-way ANOVA, N = 3.

The optimal doses of anti-IgM were tested by WST-8 assay after 48-hour treatment. All tested doses significantly reduced proliferative activities of Ramos cells (**Figure 2A**) and the intermediate dose (2 μg/ml) was selected for the following experiments. Long-term treatment (24 to 48 hours) significantly altered the activation of survival proteins AKT and p65 (**Figure 2B**), with the activation of caspase 3 activity and reduction of proteins involved in cell cycle (**Figure 2C**). No cell cycle arrest proteins (p21 and p27) were identified in the Ramos cells (**Figure 2C**). These results indicated that anti-IgM could promote cell death by suppressing expressions of survival and cell cycle proteins.

**Figure 3:**
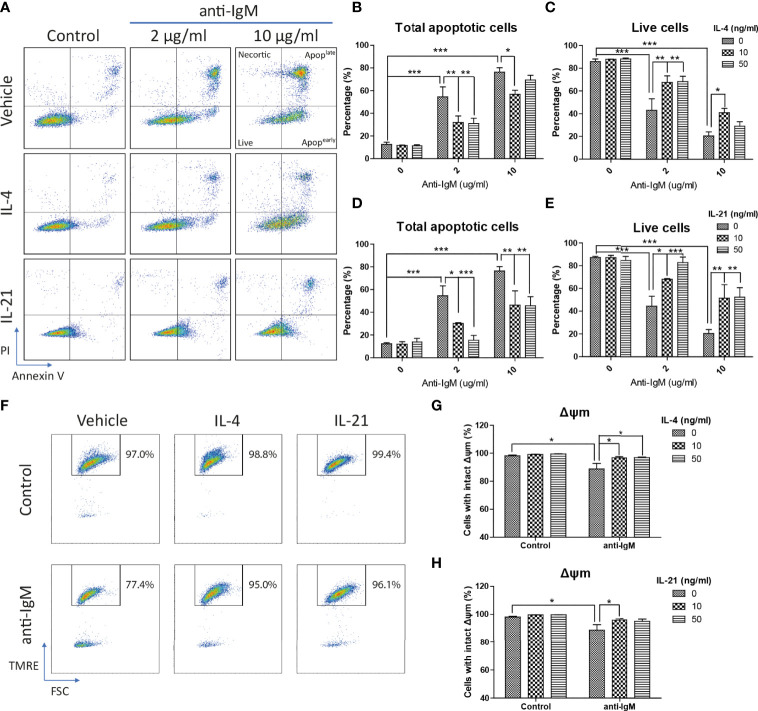
IL-4 and IL-21 rescued anti-IgM induced apoptosis and mitochondrial dysfunction. **(A)** Representative images showed that IL-4 and IL-21 suppressed anti-IgM induced early (bottom right quadrant, AV+PI-) and late (top right quadrant, AV+PI+) apoptosis, and shifted the cells back to live population (lower left quadrant, AV-PI-). Quantification showed that **(B, C)** IL-4 significantly reduced apoptotic population in both concentrations while **(D, E)** IL-21 significantly reduced apoptotic population in a dose dependent manner. **(F)** Representative images showed that IL-4 and IL-21 reversed anti-IgM (2 µg/ml) triggered loss of Δψm in Ramos. **(G, H)** Quantification confirmed the observation from the representative images. *p < 0.05, **p < 0.01, ***p < 0.001 as compared to indicated groups by two-way ANOVA, N = 4.

Next, Ramos cells were pretreated with IL-4 and IL-21 before anti-IgM challenge for 48 hours to investigate their modulatory effects. AV/PI assay was used to quantify percentages of total apoptotic cells, in which AV^+^PI^-^ represented early apoptotic and AV^+^PI^+^ indicated late apoptotic cells. Anti-IgM shifted Ramos cells from live to apoptotic cell population in a dose dependent manner, and the application of IL-4 or IL-21 reversed the anti-IgM induced apoptosis (**Figure 3A**). No obvious necrotic cell population (AV^-^ PI^+^ cells) was identified after anti-IgM treatment. Quantification confirmed that IL-4 could shift the apoptotic cell back to live cell population similarly in both low and high concentrations (**Figures 3B, C**), while IL-21 showed dose dependent effect in reversing apoptosis of Ramos cells (**Figures 3D, E**). Comparing to IL-4 treatment, application of IL-21 at 50ng/ml promoted higher survival rate of Ramos cells during anti-IgM treatment.

### IL-4 and IL-21 Rescued Ramos Cells Through Differential Mechanisms

We then investigated the underlying mechanisms of how IL-4 and IL-21 suppressed anti-IgM induced apoptosis. The collapse of the ΔΨm was reported to associate with the anti-IgM induced apoptosis in Ramos cells and eventually lead to initial release of cytochrome c into the cytoplasm (61). As shown in the TRME staining pattern, anti-IgM significantly reduced the number of cells with intact ΔΨm and both IL-4 and IL-21 could rescue this mitochondrial dysfunction (**Figure 3F**), which the quantification confirmed the result in a statistical manner (**Figures 3G, H**).

**Figure 4:**
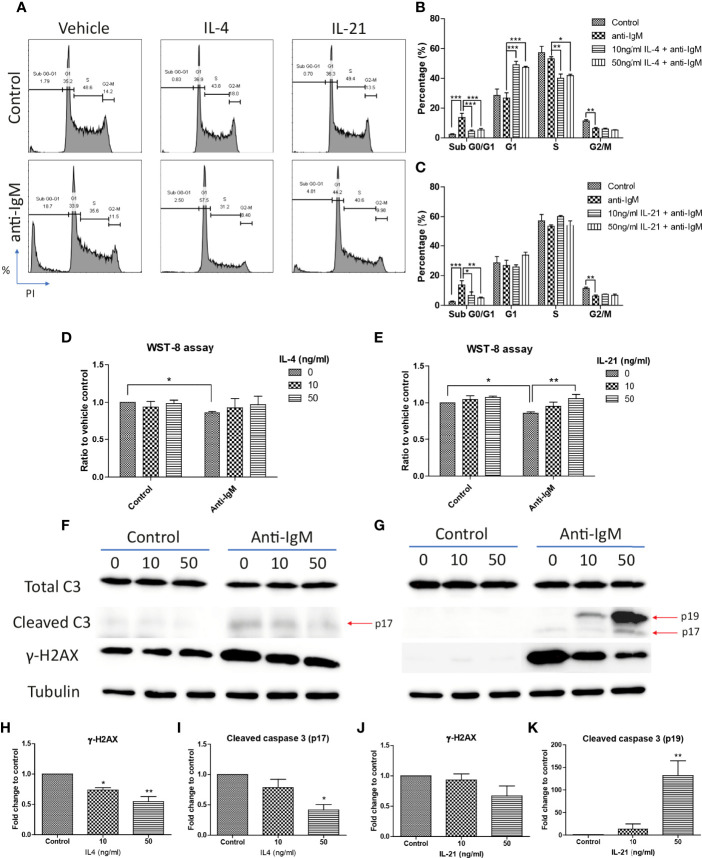
IL-4 and IL-21 rescued anti-IgM induced apoptosis by differential mechanisms. **(A)** Representative images showed that anti-IgM (2 µg/m) shifted cells from S and G2-M phases to sub G0/G1 phase, and the application of IL-4 and IL-21 reversed this deficit. Quantification showed that **(B)** IL-4 shifted the cells from sub G0/G1 to G1 phase and **(C)** IL-21 shifted cells from sub G0/G1 evenly to G1 and S phases. **(D, E)** WST-8 proliferative assays demonstrated that IL-21 (50 ng/ml) but not IL-4 re-stimulated proliferation after anti-IgM induced cell cycle arrest. **(F, G)** Representative images showed that IL-4 and IL-21 differentially modulated the levels of anti-IgM induced cleaved caspase 3 and γ-H2AX. Quantification indicated that **(H, I)** IL-4 significantly suppressed cleaved caspase 3 and γ-H2AX upregulation while **(J, K)** IL-21 lead to accumulation of intermediate isoform of cleaved caspase 3 and showed a trend to γ-H2AX suppression, under anti-IgM condition. For 4B-E, student t-test was used to compare difference between control and anti-IgM groups, while one-way ANOVA was used to determine the effects of IL-4 and IL-21 during anti-IgM treatment. For 4H-K, one-way ANOVA was used to determine the effects of IL-4 and IL-21 on suppression of cleaved caspase 3 and γ-H2AX levels as compared to anti-IgM control. *p < 0.05, **p < 0.01, ***p < 0.001. N = 4.

Cell cycle arrest is another characteristic associated with the anti-IgM induced apoptosis in B cells (45, 62, 63). We demonstrated this phenomenon in the Ramos cell model that anti-IgM significantly increased percentage of cells in sub G0/G1 phase and decreased number of cells in G2-M phase (**Figure 4A**). Both IL-4 and IL-21 significantly rescued the Ramos cells by escapes from G0/G1 phase while they differentially regulated the cell cycle reentry (**Figure 4A**). IL-4 was able to retain cells in G1 phase (**Figure 4B**) while IL-21 could evenly distribute cells into either G1 or S phases (**Figure 4C**). This phenomenon was further confirmed by the WST-8 assay that IL-21 (50ng/ml) but not IL-4 could significantly induce the proliferation rate of Ramos cells under anti-IgM conditions in a dose dependent manner (**Figures 4D, E**), suggesting IL-21 could further increase the number of abnormal B cells in the culture.

Cells accumulated in the sub G0/G1 phase are classified as apoptotic cells with DNA fragmentation and mitochondrial dysfunction (64–66). We further investigated whether the attenuation of apoptosis was related to suppression of caspase activity and DNA damage, as well as the mitochondrial dysfunction. IL-4 and IL-21 both rescued the loss of mitochondrial membrane potential (**Figures 3F-H**). Western blot analyses showed that IL-4 significantly suppressed the levels of fully mature form of cleaved caspase 3 (p17) and DNA damage marker γ-H2AX, while IL-21 could induce a trend in γ-H2AX reduction and lead to accumulation of intermediate form of cleaved caspase 3 (p19) (**Figures 4F, G**). Quantification confirmed the observation (**Figures 4H-K**). To sum up, IL-4 and IL-21 could both rescue the Ramos cells from anti-IgM induced apoptosis while the underlying mechanisms were different.

### JAK Inhibitors Demonstrated That IL-4 and IL-21 Rescued Hyperactivated Ramos Cells Through JAK/STAT Pathways

**Figure 5:**
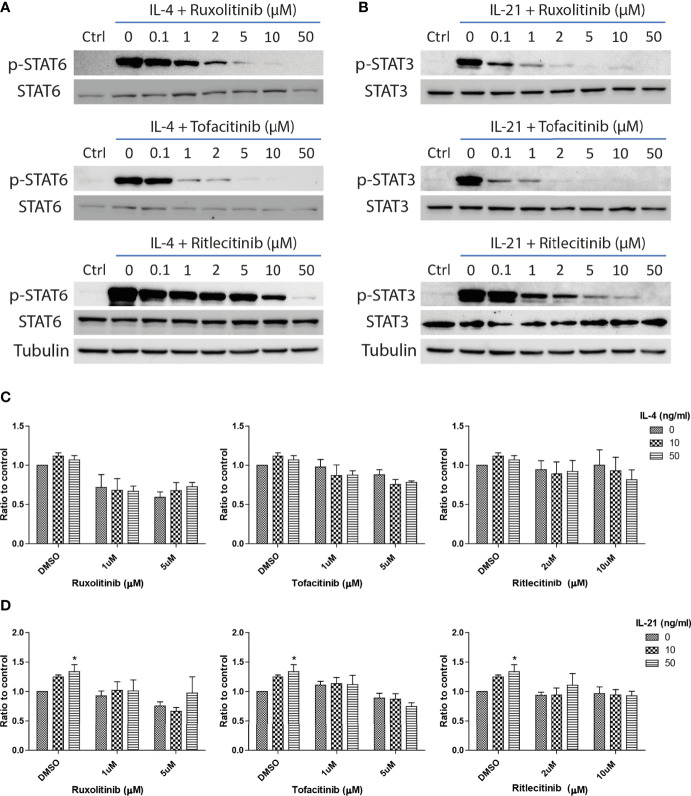
JAK inhibitors suppressed IL-21 driven proliferative activities under anti-IgM challenge. Western blot analyses demonstrated that Ruxolitinib, Tofacitinib and Ritlecitinib inhibited **(A)** IL-4 triggered STAT6 phosphorylation and **(B)** IL-21 triggered STAT3 phosphorylation in a dose dependent manner. **(C, D)** WST-8 proliferative assays showed that all tested JAK inhibitors suppressed IL-4 and IL-21 rescues of anti-IgM arrested proliferation in Ramos cells. All groups shown in the figures were treated with 1 µg/ml anti-IgM for 48 hours. *p < 0.05 as compared to indicated DMSO control group by two-way ANOVA, N = 3.

IL-4 and IL-21 could stimulate multiple downstream signaling pathways, including IRS2, Ras/MEK/ERK, PI3K/AKT/mTOR and JAK/STAT, to stimulate proliferation, activation and immune functions in the cells within the peripheral and central immune systems (67–69). As STAT6 activation is related to the survival of autoreactive B cells (70), we explored the significance of JAK/STAT pathway in the rescue of anti-IgM induced cell death through administration of JAK inhibitors. Ruxolitinib (JAK1/JAK2), Tofacitinib (JAK1/JAK3) and Ritlecitinib (JAK3 selective) were selected in the current study based on their clinical efficacies in treating lymphoma and autoimmune diseases. Ruxolitinib and Tofacitinib were approved by FDA to treat chronic graft-versus-host disease and rheumatoid arthritis, while Ritlecitinib is under clinical phase 3 trial to treat alopecia. The optimal doses of those inhibitors were first determined by their effects in suppressing major STAT phosphorylation. All three inhibitors significantly inhibited major STAT phosphorylation in the presences of IL-4 (**Figure 5A**) and IL-21 (**Figure 5B**) in a dose response manner. Low dose (1μM for Ruxolitinib and Tofacitinib, 2μM for Ritlecitinib) and high dose (5μM for Ruxolitinib and Tofacitinib, 10μM for Ritlecitinib) were selected for the following experiments.

**Figure 6:**
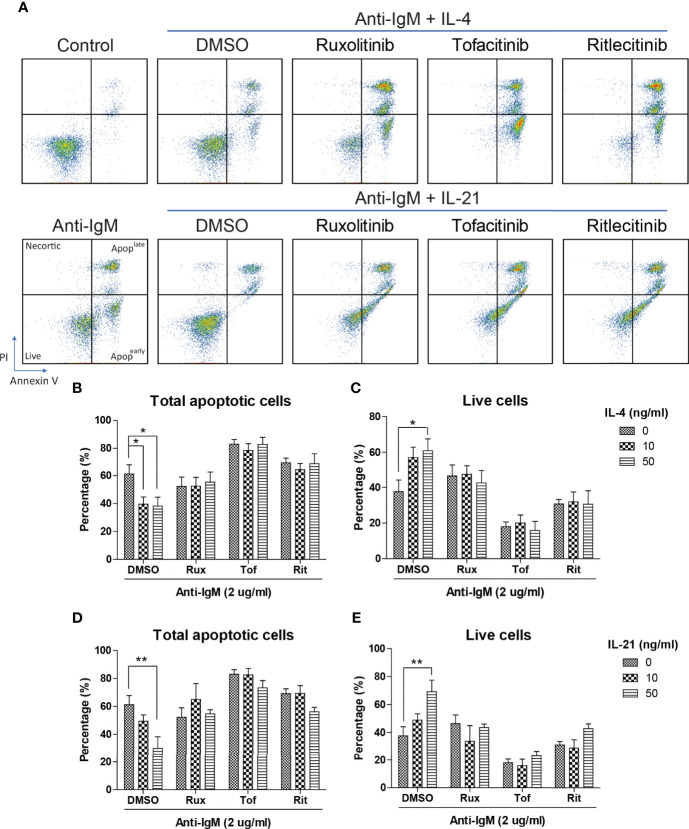
JAK inhibitors suppressed IL-4 and IL-21 rescues of B cell apoptosis. **(A)** Representative images showed that all JAK inhibitors suppressed IL-4 and IL-21 effects in suppressing apoptosis induced by anti-IgM challenge. Quantification showed that application of three JAK inhibitors re-induced apoptotic population and reduced live cell population even in the presence of IL-4 **(B, C)** and IL-21 **(D, E)** treatment. All groups shown in the figures were treated with 2 µg/ml anti-IgM for 48 hours. **p < 0.01,*p < 0.05 as compared to indicated DMSO control group by two-way ANOVA, N = 4.

**Figure 7:**
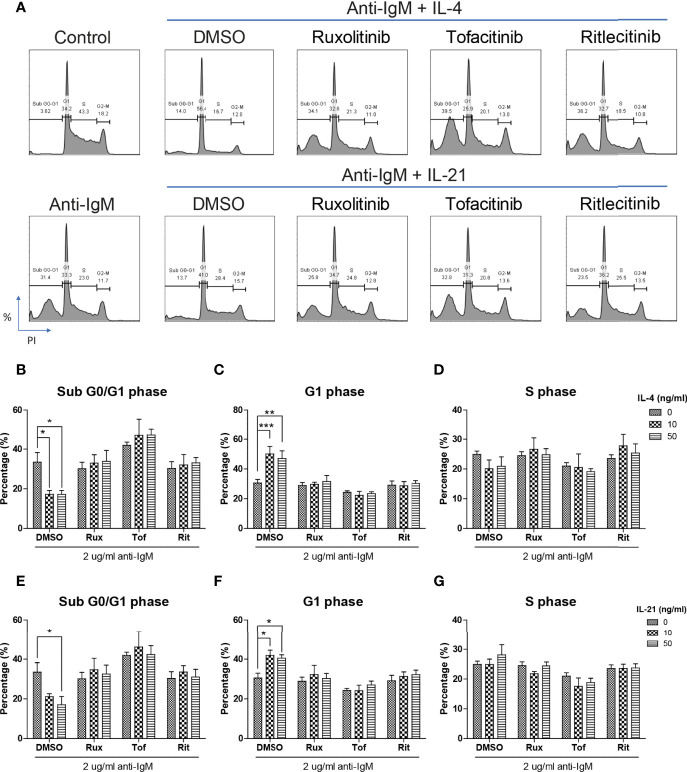
JAK inhibitors attenuated IL-4 and IL-21 rescues of cell cycle arrest and DNA damage. **(A)** Representative images showed that all JAK inhibitors suppressed IL-4 and IL-21 effects in repairment of DNA damage and cell cycle reentry. **(B-D)** Quantification showed that application of three JAK inhibitors suppressed IL-4 driven escape from sub G0/G1 and maintenance in G1 phase in Ramos cells. **(E-G)** Statistical analyses also demonstrated that all JAK inhibitors suppressed IL-21 driven escape from sub G0/G1 and cell cycle reentry in Ramos cells. All groups shown in the figures were treated with 2 µg/ml anti-IgM for 48 hours. ***p < 0.001, **p < 0.01, *p < 0.05 as compared to indicated DMSO control group by two-way ANOVA, N = 4.

**Figure 8:**
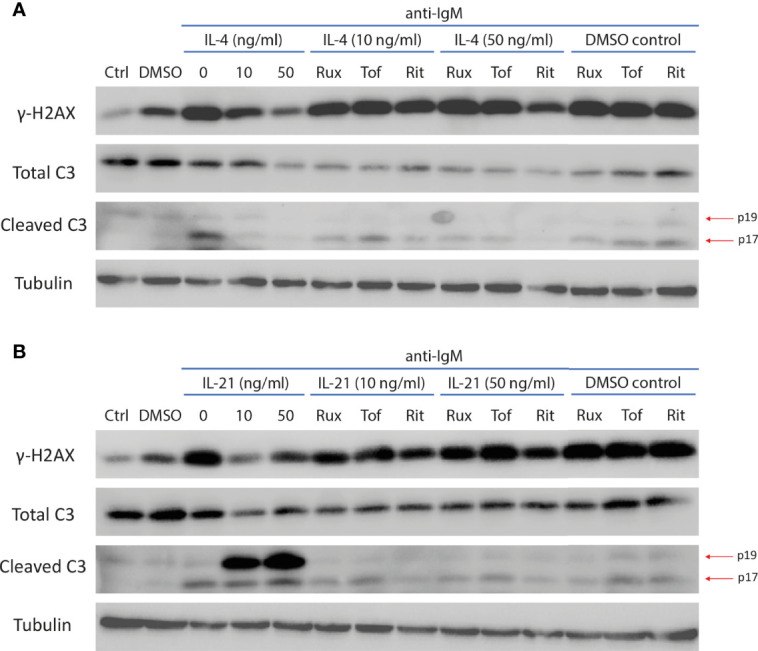
JAK inhibitors reversed IL-4 and IL-21 induced modulation of caspase 3 activity and DNA damage marker. **(A)** Representative images of western blot showing all JAK inhibitors reversed IL-4 effects in downregulating expressions of mature cleaved caspase 3 (p17) and γ-H2AX during anti-IgM stimulation. **(B)** Representative images of western blot showing all JAK inhibitors reversed IL-21 effects in suppressing expression of γ-H2AX and intermediate cleaved caspase 3 (p19) accumulation during anti-IgM stimulation. The dose of anti-IgM was 2 µg/ml and cells were treated for 24 hours.

The inhibitory effects of JAK inhibitors were first tested in the WST-8 assay. Similar to the findings from **Figures 4D, E**, IL-21 could significantly increase proliferative activities of anti-IgM treated Ramos cells while the application of JAK inhibitors blocked this effect (**Figure 5D**). IL-4 showed a trend to promote proliferation and JAK inhibitors also attenuated this effect (**Figure 5C**). The effects of JAK inhibitors were further investigated *via* AV/PI assay and cell cycle analysis. Application of three JAK inhibitors blocked IL-4 and IL-21 rescues of anti-IgM induced apoptosis (**Figure 6A**) and quantification confirmed the observation (**Figures 6B-E**). Similar results were observed in cell cycle analysis that all JAK inhibitors blocked modulatory effects of IL-4 and IL-21 in shifting cells from sub G0/G1 back to either G1 or S phases (**Figures 7A-G**). The above functional changes were consistent to the re-induction of cleaved caspase 3 and γ-H2AX, which all JAK inhibitors reversed IL-4 and IL-21 effects in modulating caspase 3 activities and downregulating γ-H2AX expression (**Figures 8A, B**). These findings demonstrated that JAK inhibitors were with potency in the order of Tofacitinib> Ruxolitinib> Ritlecitinib. To sum up, IL-4 and IL-21 mainly suppressed the anti-IgM detrimental effects through the activation of JAK/STAT pathway, which might serve as the potential therapeutic target to treat autoimmune diseases associated with hyperreactive B cell pathology.

### IL-21 Suppressed Anti-IgM and CD40L Induced Cell Death in Purified B Cells

**Figure 9:**
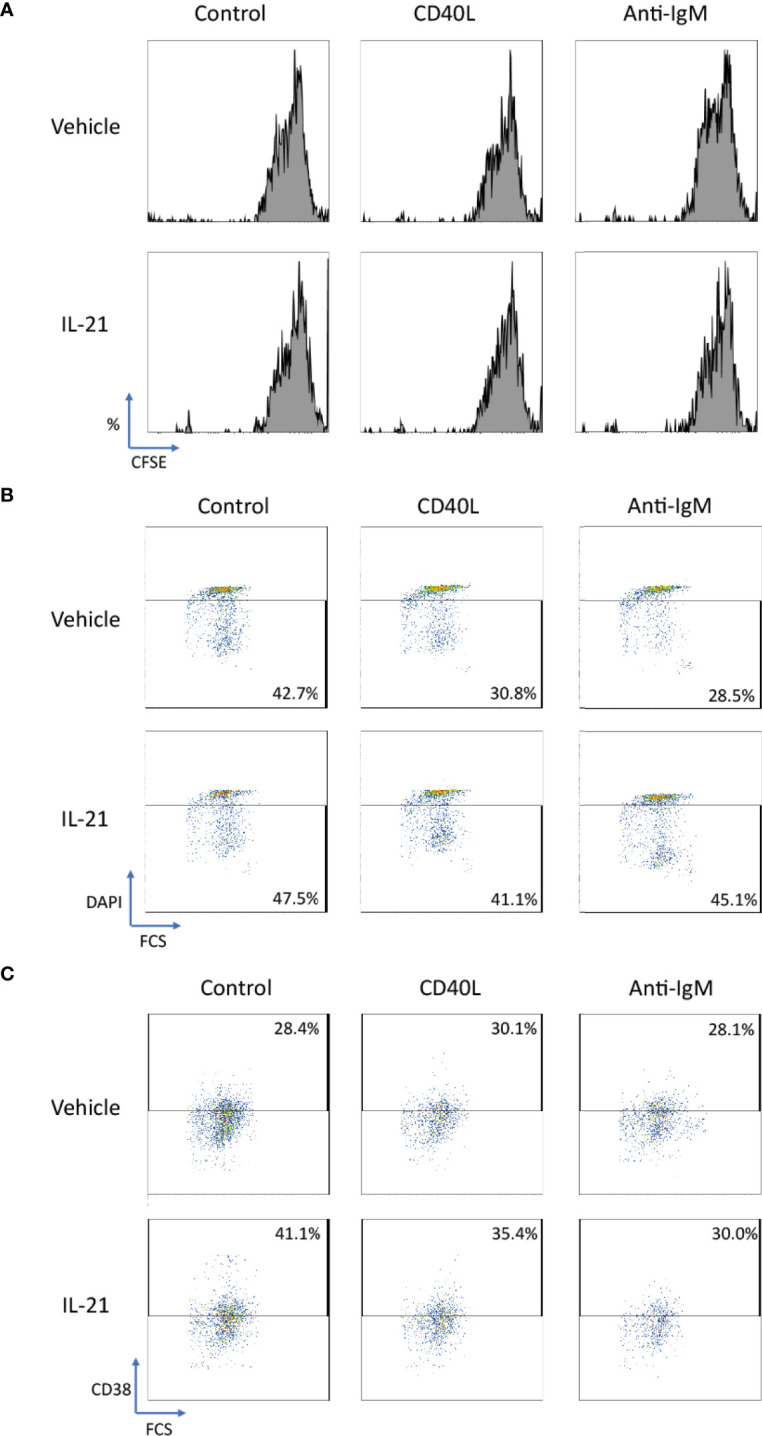
IL-21 rescued CD40L and anti-IgM induced cell death in human primary B cells. **(A)** Representative graphs of CFSE labelling cells showed no differentiation was induced by any stimulatory factors in 3 days. **(B)** Representative graphs showed that IL-21 could suppress cell death induced by CD40L and anti-IgM in terms of increased number of DAPI- cells. **(C)** Representative graphs showed that IL-21 was able to induce CD38+ B cell differentiation both in the absence and presence of CD40L.

We further extended the above studies to primary human B cells to conclude whether IL-21 induced similar beneficial effects in normal B cells. CD40L and anti-IgM were chosen as co-stimulatory factors to activate human B cells as reported in literatures (54, 71–74). Although no proliferative induction was observed in IL-21 treated groups based on CFSE staining pattern (**Figure 9A**), IL-21 potentially suppressed both CD40L and anti-IgM induced cell death (**Figure 9B**) in terms of increased population of DAPI- cells and contributed to B cell differentiation into CD38+ plasma cells (**Figure 9C**). These preliminary results demonstrated that IL-21 induced some of the beneficial effects as observed in Ramos B cells and triggered B cell differentiation as reported in literature (19).

## Discussion

The current study suggests how IL-4 and IL-21 contribute to the survival of hyperreactive B cells and the impairment in the B cell tolerance checkpoint. Numerous autoimmune diseases are B-cell mediated, characterized in the formation of autoantibodies, and/or survival of autoreactive B cells; B-cell targeted therapy such as the depletion of circulating B cells has demonstrated therapeutic success in treating these B-cell mediated diseases (11, 75). B cell tolerance checkpoints happen in both central and peripheral systems (10–12, 76, 77). Central tolerance is mediated by clonal deletion, anergy and receptor editing in the bone marrow while peripheral tolerance happens when the immature B cells expressing BCRs with low affinity for self-antigens escape central tolerance (75, 77). Those autoreactive B cells are further eliminated through anergy, clonal deletion and irresponsiveness to respective T helper cells during maturation in spleen and activation in the germinal centers within spleen and lymph nodes as a result of peripheral tolerance (75). Although there remain small fractions of autoreactive B cells present in the bloodstream of healthy individuals, they lack the abilities to enter the germinal centers and produce high affinity auto-antibodies to self-antigens (77–79), presenting minimal or no risks in the elicitation of autoimmune diseases.

The mechanisms of central and peripheral tolerance checkpoints are well documented. Autoreactive B cells can arise from deficiency in multiple tolerance checkpoints, leading to the manifestation of variety of autoimmune diseases. For example, nuclear antigen-specific autoreactive B cells in SLE patients appeared to arise from impairment in the process of anergy formation at the peripheral tolerance checkpoint as those autoreactive cells were derived mostly from the naive B-cell subset (80, 81). In RA patients, it was proposed that autoreactive B cells were formed as a result of either defective functions or deficiency of T_FH_ cells (82). More evidence has demonstrated that autoreactive B cells from different etiological sources would express differential phenotypes as observed in RA, SLE and SS patients (83–86), suggesting targeting differential genes/proteins in repairing deficiencies or defects in multiple tolerance checkpoints would have different therapeutic efficacies for treating different B-cell mediated autoimmune diseases.

We speculate that blockade of IL-4 and IL-21 upregulation serves as a common strategy by repairment of the tolerance checkpoints to trigger apoptosis of autoreactive B cells during autoimmune diseases. However, autoreactive B cells refer only to a small fraction in the whole B cell population, it is therefore difficult to detect and study the rare disease-associated antigen-specific B cells in patients (87). Blanket elimination of B cells such as using B-cell depleting anti-CD20 antibodies, albeit clinically efficacious, poses concerns over drug safety as cases of infection and malignancy were observed (88, 89). There are unmet medical needs to develop more selective and safer treatment modalities to target different autoimmune diseases. By employing anti-IgM-crosslinked BCR activation on Ramos cells to mimic B cell hyperactivation, this serves as a platform for studying potential targets with therapeutic potential for treating autoimmune diseases (45, 50, 62, 90–92). We demonstrated that Ramos cells could only respond to IL-4 and IL-21 stimulation in terms of major STAT protein phosphorylation and modulation of STAT driven gene expressions (**Figure 1**); the results were consistent with the findings of others, corroborating the contribution of these cytokines (IL-4 and IL-21) to transcriptional regulation and survival in B cells (16–21). Ramos cells that were treated with cross-linking anti-IgM for 48 hours exhibited reduced proliferation and increased apoptosis, reminiscent to how autoreactive B cells were eliminated through the tolerance checkpoints (**Figures 2** and **3A**). As the mechanisms of how IL-4 could promote abnormal active B cell function/survival during disease conditions were well documented (43–48), we would like to focus on comparing how IL-4 and IL-21 could modulate the homeostasis of hyperactivated Ramos cells. Apoptosis, cell cycle arrest, proliferation, DNA damage and mitochondrial dysfunction were investigated in Ramos cells after cytokine and anti-IgM treatment. IL-4 was able to reverse anti-IgM induced apoptosis, DNA damage and mitochondrial dysfunctions by suppressing cleaved caspase 3 activity and expression of DNA damage marker γ-H2AX (**Figures 3** and **4**). These data demonstrated that IL-4 could potentially break B cell tolerance checkpoint and promote survival of autoreactive B cells as previously reported (44, 45, 93, 94). IL-21, on the other hand, was shown to reverse anti-IgM induced mitochondrial dysfunction and apoptosis through different routes. We demonstrated that IL-21 triggered the accumulation of intermediate p19 isoform in hyperactivated Ramos cells and induction of Ramos cell proliferation, as evaluated by WST-8, cell cycle and western blot assays (**Figures 3** and **4**). We hypothesize that IL-21 suppressed apoptosis by retaining p19 isoform in the cytosol, leading to the accumulation of p19 isoform in activated immune cells and reduction in the strength of caspase 3 activity (95, 96). The current data demonstrate that IL-21 is another important contributor to the impairment of tolerance checkpoint as suggested by other literatures (97, 98). To determine whether the suppression of IL-4 and IL-21 induced downstream pathways could serve as a strategy to treat autoimmune diseases, we used three JAK inhibitors to suppress IL-4/STAT6 and IL-21/STAT3 activation in anti-IgM-treated Ramos cells. All three inhibitors reversed IL-4 and IL-21 driven rescues of cell apoptosis, cell cycle arrest and DNA damage with potency in the order of Tofacitinib> Ruxolitinib> Ritlecitinib (**Figures 6**-**8**). These data indicate that suppression of both JAK1 and JAK3 is more effective in treating autoimmune diseases that are associated with IL-4 and IL-21 upregulation.

To further explore the feasibility of this simple platform for testing drugs in B cells, we extended the studies to primary B cells isolated from the healthy human PBMC culture. It is well known that IL-4 could further enhance anti-IgM induced proliferation through IgM maturation (48, 99). Furthermore, IL-4 could protect B cell from anti-IgM induced apoptosis through upregulation of Bcl-xL and downregulation of Bim in murine and human primary B cell cultures (44, 100). These studies showed that IL-4 is one of the main factors to increase B cell survival under anti-IgM stimulation. As a recently identified cytokine, IL-21 is capable of regulating proliferation, activation and differentiation of human B cells in the presence of co-stimulatory signals (19, 101). While IL-21 mainly drive B cell apoptosis in the presence of LPS or CpG DNA stimulation (101), it could further induce CD40L or anti-IgM induced proliferation in both murine and human B cells (54, 74, 101, 102). More importantly, IL-21 could suppress anti-IgM induced apoptosis in murine B cells (101). In our 3-day B cell culture, we could demonstrate the similar phenomenon that IL-21 could suppress cell death induced by CD40L and anti-IgM treatments (**Figure 9**), while no proliferative induction was observed in 3 days as previously reported (74). These data are consistent to the some of the observation in Ramos B cells in the current studies.

JAK inhibitors, due to their limited selectivity, are known to exhibit higher incidences of undesirable side effects, confining its wider clinical applications (103). Instead, the more selective monoclonal antibodies targeting IL-4 and IL-21 were developed and tested in patients with autoimmune diseases, which showed significant clinical efficacies (104–106). The antibody therapies might help eliminating a selected population of autoreactive B cells with specific and unique phenotypes triggered by IL-4 and IL-21 (44, 70, 107, 108), as well as depleting the CD19^high^CXCR3^high^ B cell which is associated with poor clinical outcomes after rituximab treatment (109). Further studies are still needed to delineate the exact mechanisms of action of IL-4 and IL-21 on the B cell differentiation and survival of autoreactive B cells in primary B cell cultures in the presence of different co-stimulatory factors and the animal models of autoimmune diseases, and how antibodies against these cytokines can modulate the phenotypes of autoreactive B cells and improve the clinical outcomes of patients with autoimmune diseases.

## Conclusion

The current study provides a simple *in-vitro* platform to study the B cell tolerance checkpoints in the hyperactivated Ramos cells. IL-4 and IL-21 have been shown to break the tolerance checkpoints and promote differential phenotypes of the rescued cells. Blockade of IL-4 and IL-21 driven JAK/STAT pathways serves as a specific therapeutic approach to eliminate autoreactive B cells in the patients with autoimmune diseases. The antibodies targeting a combination of cytokines might provide more selective and safer approaches to target autoimmune diseases in the future.

## Data Availability Statement

The raw data supporting the conclusions of this article will be made available by the authors, without undue reservation.

## Author Contributions

CWH wrote and prepared the first draft of article. SOL reviewed the manuscript and proofread the manuscript. CWH and WCW performed the experiments and analyzed the data. All authors contributed to the article and approved the submitted version.

## Funding

This work was supported by the private funding from SinoMab BioScience Limited.

## Conflict of Interest

Author CH, WW, and SL were employed by the company SinoMab BioScience Limited.

This study received funding from SinoMab BioScience Limited. The funder was involved in the study design, collection, analysis, interpretation of data, the writing of this article and the decision to submit it for publication.

## Publisher’s Note

All claims expressed in this article are solely those of the authors and do not necessarily represent those of their affiliated organizations, or those of the publisher, the editors and the reviewers. Any product that may be evaluated in this article, or claim that may be made by its manufacturer, is not guaranteed or endorsed by the publisher.
